# Back and forth: History of and new insights on the vertebrate lymphatic valve

**DOI:** 10.1111/dgd.12757

**Published:** 2021-11-16

**Authors:** Masahiro Shin, Nathan D. Lawson

**Affiliations:** ^1^ Department of Molecular, Cell, and Cancer Biology University of Massachusetts Medical School Worcester Massachusetts USA

**Keywords:** lymphatic system, lymphatic valve, lymphatic vessel, zebrafish

## Abstract

Lymphatic valves develop from pre‐existing endothelial cells through a step‐wise process involving complex changes in cell shape and orientation, along with extracellular matrix interactions, to form two intraluminal leaflets. Once formed, valves prevent back‐flow within the lymphatic system to ensure drainage of interstitial fluid back into the circulatory system, thereby serving a critical role in maintaining fluid homeostasis. Despite the extensive anatomical characterization of lymphatic systems across numerous genus and species dating back several hundred years, valves were largely thought to be phylogenetically restricted to mammals. Accordingly, most insights into molecular and genetic mechanisms involved in lymphatic valve development have derived from mouse knockouts, as well as rare diseases in humans. However, we have recently used a combination of imaging and genetic analysis in the zebrafish to demonstrate that valves are a conserved feature of the teleost lymphatic system. Here, we provide a historical overview of comparative lymphatic valve anatomy together with recent efforts to define molecular pathways that contribute to lymphatic valve morphogenesis. Finally, we integrate our findings in zebrafish with previous work and highlight the benefits that this model provides for investigating lymphatic valve development.

## Graphical abstract

Discovery of lymphatic valve in zebrafish. The valve is structurally and genetically conserved.

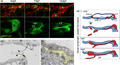

## 1 INTRODUCTION

**FIGURE 1:**
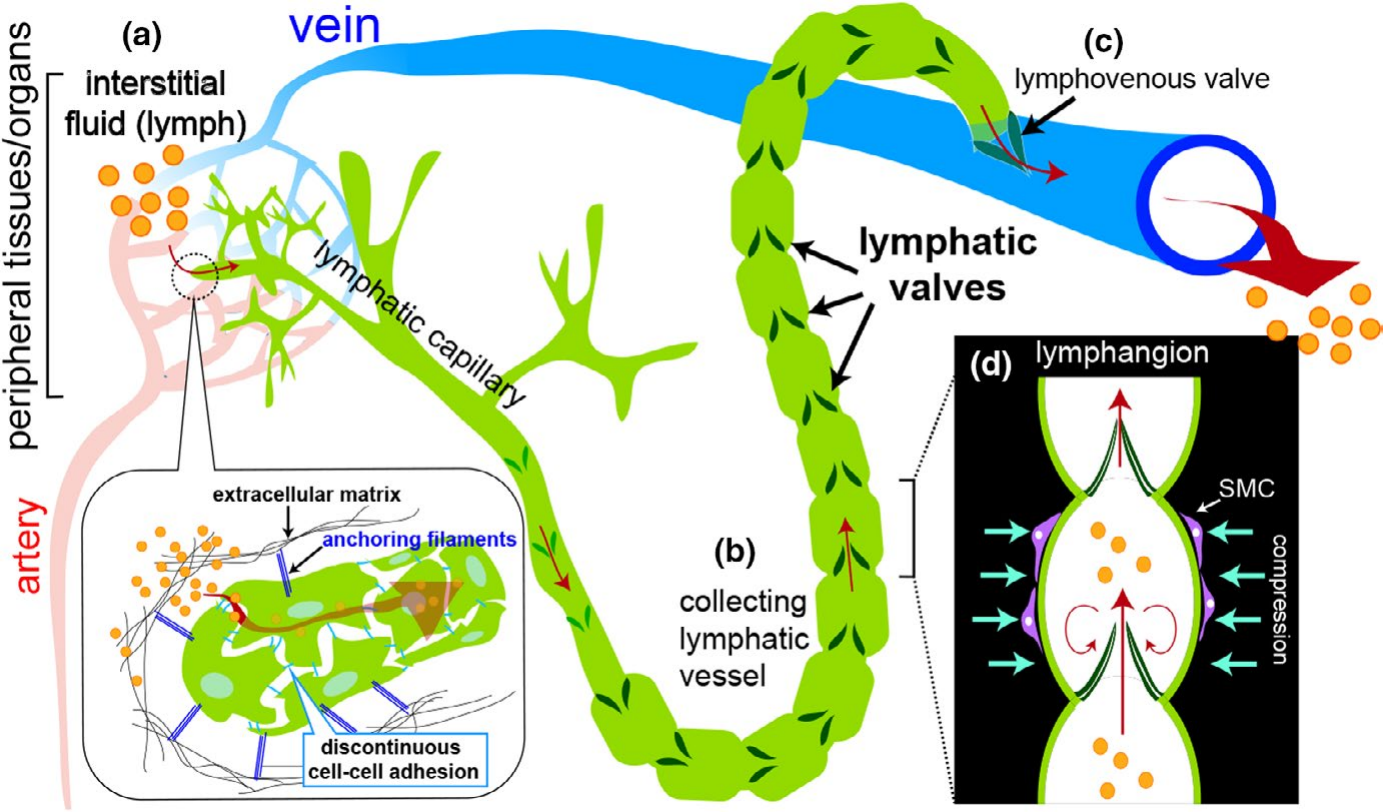
Schematic of fluid flow in the lymphatic system. (a) Interstitial fluid (referred to as lymph, orange circles) containing waste, small molecular lipoproteins and plasma leak from blood capillaries and are absorbed into lymphatic capillaries through discontinuous cell‐cell junctions. Here, lymphatic endothelial cells are held in place via anchoring filaments (blue lines) attached to surrounding extracellular matrix (black lines). (b) Lymph is transported from lymphatic capillaries into collecting lymphatic vessels where unidirectional lymph flow is maintained by numerous intraluminal lymphatic valves (dark green). (c) Collecting lymphatic vessels make direct connections with a vein through a lymphovenous valve to return fluid back to the circulatory system. (d) The region between successive lymphatic valves within the collecting vessel is referred to as a lymphangion. Collecting vessels exhibit smooth muscle cell coverage (SMC, purple cells) to aid in propulsion, while the bulk of flow is stimulated by the contraction of neighboring skeletal muscles

The lymphatic system is a network of vessels lined by a monolayer of specialized endothelial cells (lymphatic endothelial cells, or LECs). During vertebrate embryogenesis, selected endothelial cells in the jugular and cardinal veins differentiate into LECs and subsequently separate from the circulatory system to give rise to a primitive lymphatic vessel network (Koltowska et al., 2013). Further maturation of lymphatic vessels results in a continuous interconnected network broadly subdivided into lymphatic capillaries and collecting lymphatic vessels. Lymphatic capillaries lie in close association to the peripheral blood vessels, called capillary beds, of the circulatory system and are supported by attachment via anchoring filaments to the surrounding extracellular matrix (Figure 1a). Endothelial cells within lymphatic capillaries exhibit discontinuous cell‐cell adhesion, which facilitates the easy absorption of protein‐ and lipid‐rich interstitial fluid that leaks from the capillary beds of the circulatory system. This fluid, referred to as lymph, is then transported into collecting lymphatic vessels (Figure 1b) and returned to the circulatory system through a direct connection to a vein. A valve at the lymphatic/venous interface, called the lymphovenous valve allows fluid passage but prevents entry of blood cells into the lymphatic system (Figure 1c). Thus, the central function of the lymphatic system is to maintain fluid homeostasis, although it also plays important roles in fat absorption in the intestine and immune cell surveillance of lymphocytes (e.g. dendritic cells and T‐cells) traveling through the lymphatic system together with their residing in lymph nodes (Alitalo, 2011).

The vessels of the lymphatic system are blind‐ended, unlike the circulatory system, which is a closed loop comprising blood vessels and a heart (Alitalo, 2011). The mammalian lymphatic system lacks an analogous organ to provide pulsatile force to the fluid within the lymphatic vessels, relying instead on the movement of the adjacent musculoskeletal system. By contrast, several species of amphibians, birds, and reptiles possess lymph hearts, which can actively drive pulsatile flow through the lymphatic system (reviewed in Hillman et al., 2021). Regardless of the means of lymph propulsion, valves within lymphatic vessels are also important to maintain unidirectional flow. Lymphatic valves are composed of two semilunar leaflets formed from specialized LECs bound to a central extracellular matrix core. These intraluminal valves are formed in collecting lymphatic vessels in mammals (Figure 1b), or in close association with the lymphatic heart in amphibians (Kampmeier, 1969). In mammalian collecting lymphatic vessels, valves form at regular intervals, giving rise to discrete compartments (referred to as lymphangion) through which lymph traverses as it is pushed along by musculoskeletal movement (Figure 1d). Support for an essential role for valves in lymphatic function and fluid homeostasis derives from numerous mouse knockouts that preferentially block valve morphogenesis (see below). In these cases, mouse embryos present with accumulation of interstitial fluid, referred to as lymphedema, due to a failure in unidirectional lymph transport.

Over the past two decades, the zebrafish has become established as a model to study development of the circulatory and lymphatic systems (Koltowska et al., 2013; Nakajima et al., 2021). By taking advantage of its transparent and externally developing embryos, researchers have been able to directly visualize endothelial cell behaviors in vivo. Combining high content live imaging techniques with a wide range of genetic approaches has enabled identification of new genes and pathways required for the development of blood and lymphatic vessels (Koltowska et al., 2013; Matsuoka & Stainier, 2018). Consequently, the zebrafish model has yielded numerous novel insights into the cellular and molecular mechanisms that contribute to circulatory and lymphatic system development (Koltowska et al., 2013; Nakajima et al., 2021). Despite the extensive anatomical characterization of the lymphatic system in zebrafish embryos and other related species, valves had not been identified or characterized in teleost fish until our recent discovery. Below we provide a historical overview of previous studies concerning lymphatic valves, along with a summary of our recent findings.

## 2 HISTORICAL PERSPECTIVE

Among the earliest contributors to the study of the lymphatic system were Hippocrates (450–380 B.C.), Aristotle, and Galen, who provided the first rudimentary descriptions of lymphatic vessels, though their prospective function was unclear at the time of their observations (Irschick et al., 2019; Natale et al., 2017). Among these early studies, Hippocrates noted lymphatic vessels as “containing white blood”, likely due to the presence of lipid‐rich lymph. This observation underscored the challenge in visualizing lymphatic vessels especially in postmortem samples, as the opaque appearance of lymph is not uniform, limiting comprehensive analysis without other means of imaging. It is likely that this technical hurdle caused characterization of the lymphatic system to lag that of the circulatory system, which was more easily visualized by the presence of red blood cells. Indeed, the pioneering work of William Harvey, which provided functional definitions for arteries and veins, was published in 1628 (Aird, 2011) more than 100 years before comparable characterization of the lymphatic system (see below).

Given the limitations on visualizing lymphatic vessels, initial anatomical descriptions dating from the 16th and 17th century initially focused on lacteal lymphatic vessels in the intestine, which carried opaque lipid‐rich lymph (Irschick et al., 2019; Natale et al., 2017). Indeed, Gaspare Aselli re‐discovered the lacteal lymphatic vessel network in 1622 following vivisection of a canine immediately after feeding, when lipid absorption into these vessels would be at its peak (Irschick et al., 2019). He also was able to visualize valves within lacteals, although he failed to recognize the connection of these vessels to a systemic lymphatic network. Lymphatic valves in the thoracic duct were independently noted by French anatomist Jean Pecquet and Dutch anatomist Jan van Horne in 1651 and 1652, respectively, the former of which was able to trace the path of lymph flow from lacteals into the thoracic duct, and into the subclavian vein (Irschick et al., 2019; McDonald & Russell, 2018; Natale et al., 2017). Shortly thereafter, Niels Stensen described the existence of a lymphovenous valve and the apparent connection between the thoracic duct and subclavian vein (Natale et al., 2017). Despite these advances, lymphatic vessel function was not clearly established until the mid‐18th century, when studies were enabled by improved techniques for tissue fixation and visualization. The Dutch anatomist Frederik Ruysch pioneered the use of mercury sulfide and glycerol for fixing and visualizing lymphatic vessels leading to a detailed description of lymphatic valves (Ijpma & van Gulik, 2013). Ruysch's work also revealed initial insights into the direction of lymph flow. Soon thereafter, William Hunter provided a comprehensive description of lymphatic capillaries and collecting vessels and their important role in draining fluid from interstitial tissue (McDonald & Russell, 2018). From his observations, Hunter proposed that lymphatics are clearly separate from the circulatory system and that valves are important for maintaining unidirectional flow in the absence of a source for pulsatile force (i.e. a heart). In subsequent collaborations with William Hunter, William Hewson provided an extensive description of lymphatic vessels in numerous non‐mammalian species, including birds and bony fishes (teleosts; Hewson & Hunter, 1769). However, Hewson failed to identify lymph nodes or valves in teleosts, possibly due to their smaller size.

The advent of higher magnification imaging techniques in the 20th century, along with the application of more refined serial imaging of live samples, provided increasingly detailed insights into the ultrastructure of lymphatic valves in mammals (for example see, Vajda & Tomcsik, 1971). Application of transmission and scanning electron microscopy revealed that lymphatic valves comprise two bicuspid leaflets, aligned in a manner consistent with promoting unidirectional flow (see below, Albertine et al., 1982; Bazigou et al., 2009). In the early 2000s, studies began to focus on the mouse lymphatic valve, bringing to bear a genetic model that would allow discovery of essential genes required for lymphatic valve morphogenesis (Koltowska et al., 2013). Subsequent work over the past decade has revealed numerous genetic pathways required for lymphatic development, as well as the importance for physiological signals, such as disturbed flow within lymphatic vessels. Importantly, insights from mouse studies have revealed that several congenital human syndromes associated with lymphedema were caused by genes required for lymphatic valve development (Koltowska et al., 2013; Petrova et al., 2004).

## 3 LYMPHATIC VALVE FORMATION IN MOUSE

Studies in the mouse have contributed to a detailed understanding of the cellular and molecular mechanisms that contribute to lymphatic valve formation. Here we provide a general overview of this process, with emphasis on selected molecular players, particularly those relevant to recent zebrafish studies. For a more detailed discussion of the molecular and cellular aspects of lymphatic valve morphogenesis, we refer readers to several excellent review articles (Balint & Jakus, 2021; Francois et al., 2021; Geng et al., 2017, 2021; Koltowska et al., 2013).

**FIGURE 2:**
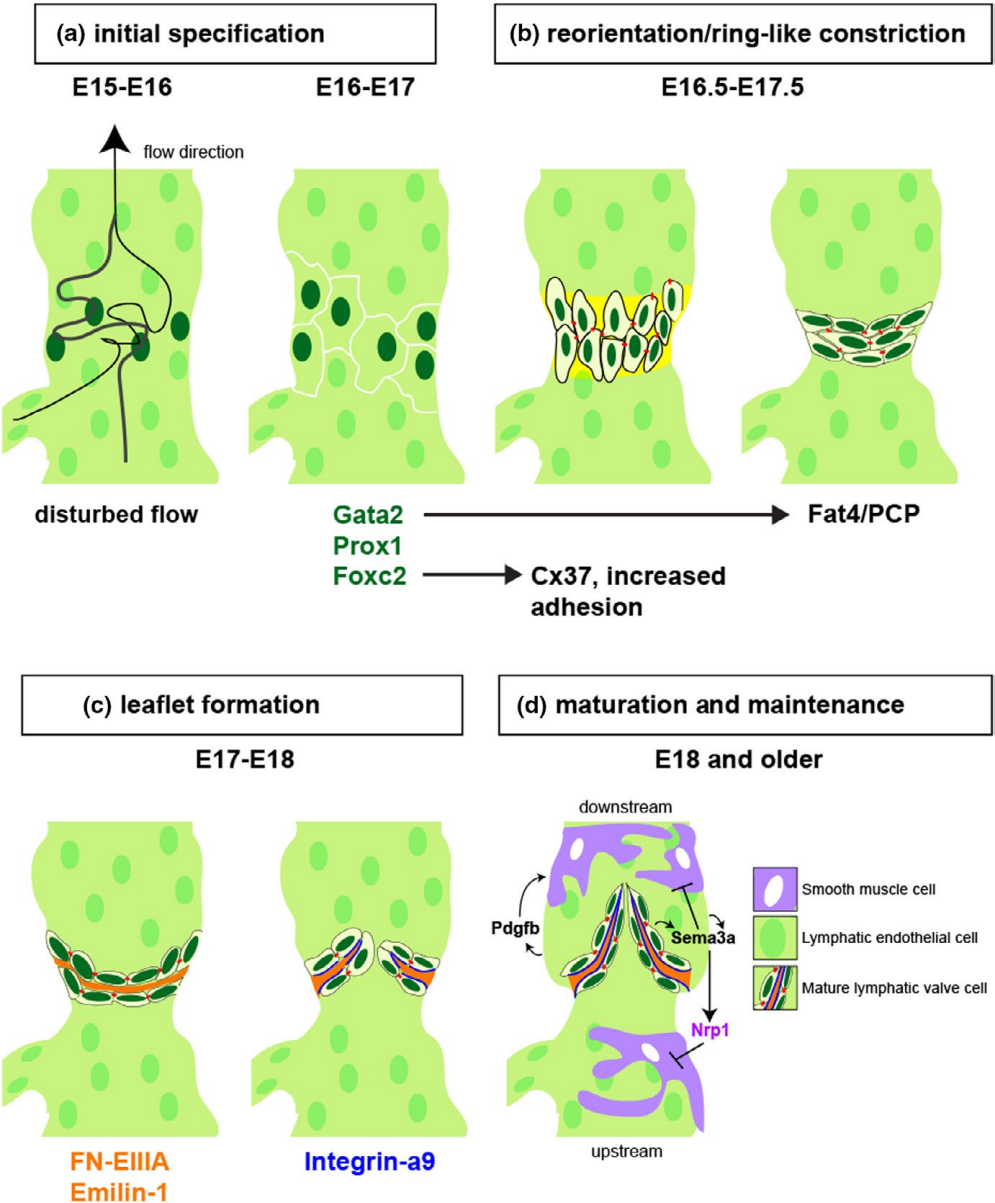
A schematic drawing of lymphatic valve development in collecting lymphatic vessel of mouse model adjusted and modified from (Koltowska et al., 2013; Tatin et al., 2013). (a) From E15~E16, disturbed lymph flow (from bottom to top) downstream from a branch point induces Foxc2 and Gata2 expression, which in turn induces Prox1 to initiate valve development at E16‐E17 (Cha et al., 2018; Kazenwadel et al., 2015; Norrmén et al., 2009; Petrova et al., 2004; Qu et al., 2015; Sabine et al., 2012; Zhou et al., 2010). (b) From E16.5 to E17.5, key transcription factors stimulated by shear stress regulate cell‐cell adhesion (e.g. Foxc2 and Cx37) and cell polarity (e.g. Gata2 and Fat4) to promote reorganization of lymphatic valve cells to form a ring‐like constriction with expression of basement membrane proteins (yellow). (c) Between E17 and E18, LvEC secrete ligands for Integrin‐a9 (orange, FN‐EIIIA and Emilin‐1) into an extracellular matrix core, and express Integrin‐a9 on their basal surface (blue lines) to promote valve leaflet extension. (d) At E18.0 or later, lymphatic valves are fully mature and collecting lymphatic vessels exhibit smooth muscle coverage (SMCs, purple). SMCs are recruited by Pdgfb, except at the valve, where they are repelled by Sema3a binding to Nrp1, a direct target of Nrp1. Subcellular types in mature lymphatic valves are color‐coded and listed

In the mouse embryo, lymphatic endothelial cells (LECs) form a largely uniform blind‐ended network of vessels by E14.5 (Koltowska et al., 2013). Soon thereafter (E15), initial specification of lymphatic valve endothelial cell (LvEC) identity is evident in the stepwise expression of several transcription factors, beginning with the zinc finger protein Gata2. Subsequently, LvECs show increased expression of the Forkhead protein Foxc2 and Prox1, the well‐known master regulator of lymphatic cell fate, which is a direct target of Gata2 (Betterman et al., 2020; Kazenwadel et al., 2012, 2015). These transcription factors are expressed in LvECs at regular intervals along the nascent collecting vessel that later correspond to the location of lymphatic valves between lymphangion segments (see Figure 1d). LvEC expression of Gata2 is initiated, in part, by oscillatory shear stress (OSS) caused by disturbed lymph flow patterns at branch points in the collecting vessel (Figure 2a; Sweet et al., 2015). OSS can activate transcription through an intronic enhancer in Gata2, providing a direct mechanistic connection between OSS mechanosensation and LvEC specification (Janardhan et al., 2017). The OSS mechanoreceptor in this context is currently unknown, although Cadherin 5 (Cdh5) and Vascular endothelial growth factor receptor‐3 (Vegfr3, also known as Flt4), the receptor for vascular endothelial growth factor c (Vegfc) may play important roles (Cha et al., 2018; Yang et al., 2019), as both interact in blood vascular endothelial cells to form a mechanosensory complex (Baeyens et al., 2015). However, whether they play a similar role to detect OSS for LvEC specification is not known.

Following specification, LvECs change their adhesive properties and acquire a cuboidal shape. LvECs then reorient themselves perpendicular to the surrounding non‐valve LECs and form a ring‐like constriction around the collecting vessel lumen (Figure 2b). These cell behaviors are largely facilitated through targets of the LvEC transcription factors described above. For example, Foxc2 induces expression of Connexin37, an endothelial‐expressed gap junction protein important for communication between adjacent LvECs (Hautefort et al., 2019). In mice, Connexin37‐deficient cells do not reorient properly and fail to form a ring‐like constriction, although earlier LvEC specification is unaffected. In parallel, Gata2 directly induces expression of *Fat4*, a component of the Planar Cell Polarity (PCP) pathway (Betterman et al., 2020; Kazenwadel et al., 2012, 2015). PCP refers to the process of directional cell organization facilitated through cell:cell contacts (Yang & Mlodzik, 2015). Fat4 is an atypical cadherin that mediates homotypic interaction between cells in the context of PCP (Figure 2b). *FAT4* mutations in humans are associated with Hennekam syndrome, a rare congenital disease characterized by lymphedema, while loss of *Fat4* in mouse prevents perpendicular rearrangement of LvECs without affecting specification.

As with initial LvEC specification, mechanosensation of lymph flow is also essential during early lymphatic valve morphogenesis. Piezo1 is a non‐selective cation transporter located on the cell surface that directly senses mechanical forces in endothelial cells. In mouse, loss of Piezo1 reduces LvEC cell‐cell adhesion leading to defects in cellular condensation and a failure to form a ring‐like constriction (Choi et al., 2019; Nonomura et al., 2018), while *PIEZO1* mutations are associated with lymphedema in humans (Fotiou et al., 2015; Lukacs et al., 2015). Interestingly, initial specification of the lymphatic valve region, as indicated by expression of Prox1, Foxc1, and Nfatc1, is unaffected by loss of Piezo1, suggesting functionally distinct mechanosensory inputs at the specification and morphogenesis stages in vivo (Nonomura et al., 2018). However, loss of Piezo1 in adult mice causes lymphatic valve regression, while PIEZO1 activation in human lymphatic endothelial cells can induce valve genes, even in the absence of OSS (Choi et al., 2019). Thus, signaling through Piezo1 likely plays dual roles in initial morphogenesis and long‐term maintenance of lymphatic valves. Downstream effectors of early valve morphogenesis are also sensitive to flow conditions. For example, Fat4 exhibits flow‐dependent membrane localization in cultured LECs, while loss of Fat4 prevents LEC polarization and orientation in response to flow.

From E17 to E18, LvECs extend into the lumen to initiate leaflet elongation (Figure 2c). This process is characterized by the expression of Integrins‐a9 (Itga9) and ‐b1 (Itgb1), which form heterodimers that bind to extracellular matrix (ECM) proteins (Humphries et al., 2006), an essential step during leaflet extension. In agreement, *Itga9* ^−/−^ mice exhibit fewer lymphatic valves with shorter leaflets than wild type siblings (Bazigou et al., 2009; Hess et al., 2014). In *Itga9* ^−/−^ lymphatic valves, the basal surfaces of cell layers are disorganized and do not fully contact the ECM core (Bazigou et al., 2009). LvECs at this stage also secrete ECM components, including Itga9/b1 ligands. Among these are a LvEC‐specific isoform of Fibronectin containing EIIIA domain (FNEIIIA), as well as Elastin Microfibril Interfacer 1 (Emilin1), and Sushi, Von Willebrand Factor Type A, EGF and pentraxin domain containing 1 (Svep1). Notably, FNEIIIA is specifically expressed in lymphatic valve leaflets while the more common Fibronectin isoform is expressed in basement membrane of all lymphatic vessels (Bazigou et al., 2009). Emilin1 is expressed in all lymphatic vessels, although it becomes pronounced in lymphatic valve leaflets, while Svep1 is expressed in mesenchyme surrounding lymphatic vessels. *FnEIIIA* ^−/−^ or *Emilin1* ^−/−^ mice exhibit reduced number of lymphatic valves in mesenteric collecting vessels (Bazigou et al., 2009; Capuano et al., 2019; Danussi et al., 2013) and leaflet extension does not proceed past the ring‐condensation stage. These phenotypes appear to be specific to valve formation, while *Svep1* ^−/−^ mice exhibit abnormal leaflet formation along with broader lymphatic developmental defects (Karpanen et al., 2017).

From E18 to P1, lymphatic valve leaflets continue to elongate while the lymphangion itself exhibits coverage with smooth muscle cells (SMC, Figure 2d). SMC coverage at collecting vessels is driven by Platelet‐derived growth factor b (Pdgfb) expressed in the LECs, which subsequently induces the recruitment of mural cells expressing the Pdgfb receptor, Pdgfrb (Wang et al., 2017). LEC‐specific deletion of *Pdgfb* eliminates SMC coverage on collecting lymphatic vessels leading to dilation and defects in pulsatile contraction. Interestingly, SMC coverage is absent at the site of the lymphatic valve in the collecting vessels (Figure 2d). This characteristic is mediated, in part, through Semaphorin/Plexin signaling, a repulsive cell guidance cue in the nervous and circulatory systems (Epstein et al., 2015). LvECs express two receptors for Semaphorin 3a (Sema3a), Neuropilin1 (Nrp1) and PlexinA1 (PlxnA1). Loss of Sema3a or PlxnA1, or deletion of the Sema3A‐binding domain in Nrp1, causes defects in valve development, without affecting LvEC specification, or earlier aspects of lymphatic development. In each case, ectopic SMC coverage is apparent at the region of malformed valves (Bouvrée et al., 2012). Interestingly, Nrp1 is a direct target of Foxc2 and loss of this transcription factor also results in ectopic SMC coverage in the collecting vessels in regions of the lymphatic valve. Thus, a Foxc2/Sema/Nrp/Plxn signaling axis appears essential to limit SMC at the region of the lymphatic valve. How SMC coverage may negatively impact valve maintenance, and how these factors may crosstalk with Pdgfb/Pdgfrb signaling to coordinate this process is currently not clear.

Following lymphatic valve morphogenesis, active signaling mechanisms continue to maintain normal lymphatic valve function and anatomy throughout postnatal and adult life. As with the earliest steps of LvEC specification and subsequent morphogenesis, physical forces from lymph flow, together with mechanosensitive pathways noted above, continue to contribute to postnatal valve maintenance. Accordingly, conditional LEC‐specific deletion of Piezo1 in adult mice leads to valve degeneration. In this context, Foxc2 is an important downstream effector of mechanosensation that is required for valve maintenance (Figure 2d). In postnatal collecting lymphatic vessels, *Foxc2* is preferentially expressed in LvECs on the downstream side of lymphatic valves, which are typically subjected to recirculating flow and OSS (Sabine et al., 2015; see Figure 2d). This observation is consistent with the induction of *FOXC2* in cultured LECs exposed to OSS (Sabine et al., 2012). In this setting, OSS induces LEC quiescence, which requires FOXC2 function. FOXC2 also acts downstream of OSS to maintain junctional integrity and cortical stress fibers in LECs (Sabine et al., 2015). Accordingly, LEC‐specific knockout of *Foxc2* mice during early postnatal stages (P4‐P8) or as late as 4 weeks of age caused degeneration of mature lymphatic valves, leading to a decrease in the numbers of valves and reduced resistance to back flow pressure. Thus, early pathways required for lymphatic valve development continue to be important to maintain valve structure and lymphatic function throughout adult life.

## 4 LYMPHATIC VALVES IN ZEBRAFISH

Over the past decade, the zebrafish has proven an excellent model for studying early development of the lymphatic system. Despite observations dating to the 17th century on the appearance of lymphatic vessels in multiple teleost species, there was initial debate as to their existence in zebrafish embryos (Ny et al., 2006). However, in 2006 Yaniv et al. used a combination of endothelial‐specific transgenic lines, time‐lapse analysis, and lymphangiography to describe a functional lymphatic system in the zebrafish (Yaniv et al., 2006). Subsequent studies confirmed the roles of conserved signaling molecules in zebrafish lymphatic development, including Vascular endothelial growth factor receptor c (Vegfc) and its receptor, Flt4, and the Prox1 transcription factor (Koltowska et al., 2015; Shin et al., 2016; Villefranc et al., 2013). Zebrafish studies have also yielded numerous insights into the earliest steps of lymphatic vessel formation (Koltowska et al., 2013). These include direct evidence supporting a venous origin of lymphatic vessels and identification of new essential genes, several of which were subsequently implicated in rare human genetic disorders associated with lymphedema (Alders et al., 2009). More recently, the development and characterization of new transgenic zebrafish lines for visualizing lymphatic endothelial cells has led to a re‐discovery of meningeal lymphatics in the brain and the importance of lymphatic vessels in revascularizing the central nervous system following injury (Chen et al., 2019). Thus, the zebrafish has provided major contributions and novel findings relating to the development of the lymphatic system. Despite these advances, there had not been reports of lymphatic valves in zebrafish, consistent with early studies from Hewson and Hunter (Hewson & Hunter, 1769). However, we recently used a combination of molecular, cellular, and genetic analyses to demonstrate that the zebrafish larval lymphatic system contains valves that are functionally essential during development. The following provides an overview of our recent findings.

### 4.1 Functional evidence for lymphatic valves in zebrafish

**FIGURE 3:**
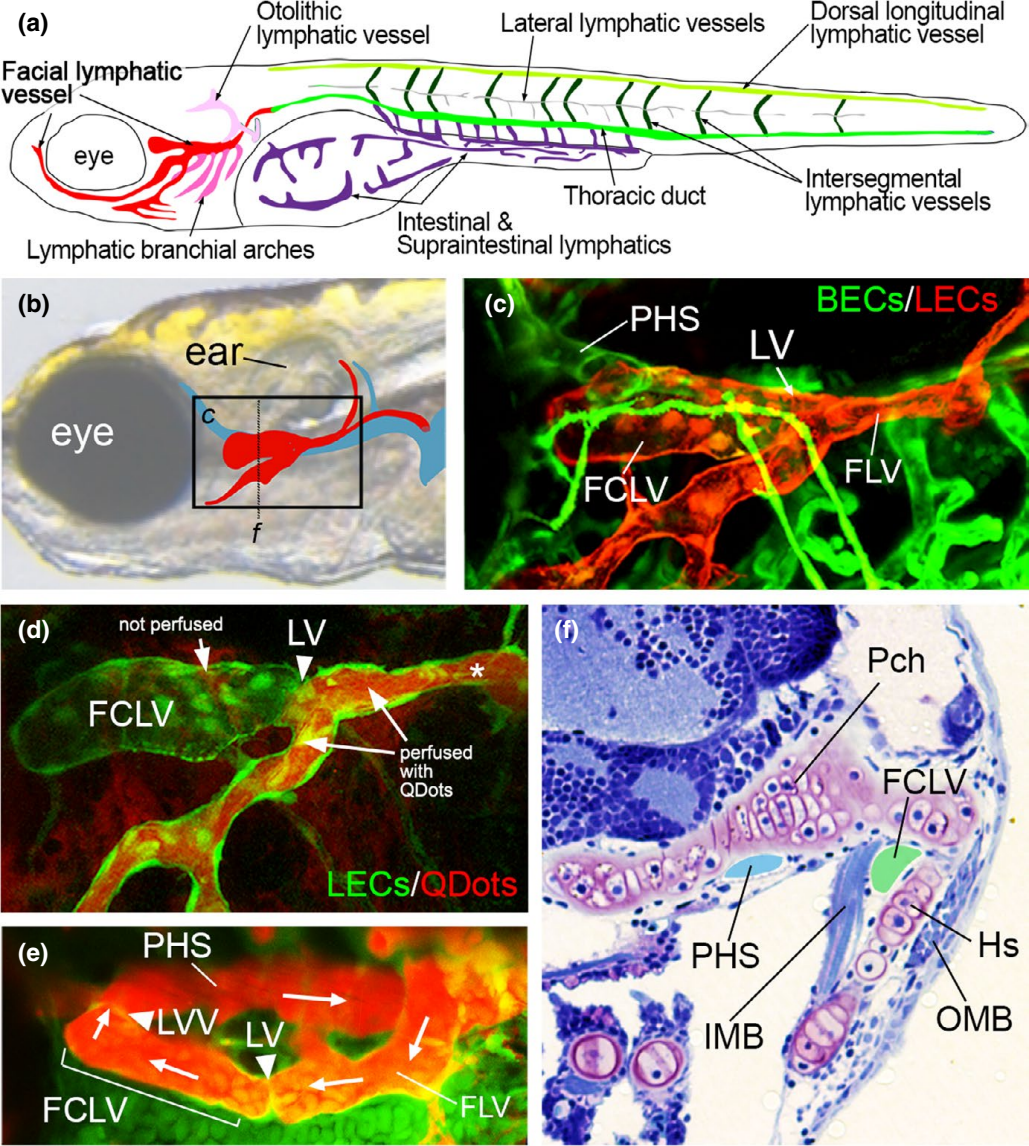
Larval zebrafish facial lymphatic vessel network. (a) Schematic drawing of main lymphatic networks of facial, lateral intestinal and trunk regions of the zebrafish larvae in lateral view. Lymphatic vessels in each network are classified and color‐coded as indicated. (b) Schematic depicting location of facial lymphatic vessel (red) and primary head sinus (blue) on transmitted light image of a zebrafish larval head. Box denotes region in (c), dotted line indicates plane of section shown in (f). (c) Confocal image of blood vessels (blood vascular endothelial cells, BECs, express green fluorescent protein) and facial lymphatic vessel endothelial cells (LECs, red). PHS, primary head sinus; LV, lymphatic valve; FLV, facial lymphatic vessel. (d) Confocal lymphangiography of FCLV and FLV in zebrafish larva at 7 dpf. LECs express green fluorescent protein. Regions with and without QDot perfusion (red) are indicated. Asterisk denotes location of QDot injection. (e) Confocal lymphangiography depicting facial lymphatic vessels and PHS. Ventral view, anterior to the left. Position of the lymphovenous valve (LVV) and LV are shown. Arrows indicate direction of flow from FLV to FCLV and back into the PHS. (f) Transverse section of trypan blue‐stained zebrafish larva. Lumens of PHS and FCLV are blue and light green, respectively. Frontal view, dorsal is up. HS, hyosymplectic cartilage; IMB, inner muscle bundle; OMB, outer muscle bundle; Pch, parachordal cartilage. Image courtesy of Sumio Isogai. Schematic illustration (a) is modified from Okuda et al. (2012), with permission. Schematic and images (b–d) are re‐used from Shin et al. (2019), while (e) from Shin et al. (2016) with permissions

Similar to mouse, zebrafish LECs typically differentiate from veins to form lymphatic networks during embryonic development. In the trunk, LEC progenitors arise from the posterior cardinal vein as lymphangioblasts following formation of major trunk blood vessels and onset of blood circulation. Lymphangioblasts migrate dorsally along intersegmental arteries and rostrocaudally along the horizontal myoseptum to give rise to intersegmental and lateral lymphatic vessels, respectively (Figure 3a). Lymphatic progenitor from the lateral lymphatics migrate ventrally to give rise to the thoracic duct (Figure 3a; review in Koltowska et al., 2013). Lymphatic progenitors from the posterior cardinal vein similarly migrate ventrally to initiate formation of the intestinal lymphatic network (Figure 3a; Hen et al., 2015). In parallel to the trunk lymphatic network, lymphatic progenitors in the head region arise from the venous primary head sinus (PHS; equivalent to jugular vein in Figure 5) and common cardinal vein to form a facial lymphatic network (Figure 3a–d). Interestingly, genetic studies using different Flt4 alleles suggest that trunk lymphatic vessels are dispensable for lymphatic function during at larval stages, while facial lymphatic are essential (Shin et al., 2016).

In the course of previous studies, we applied lymphangiography with fluorescent QDots to visualize the facial lymphatic network in larval zebrafish (Shin et al., 2016). For this purpose, we injected QDots into the caudal branch of the facial lymphatic vessel (Figure 3d) and often noted perfusion was blocked at a consistent location in the facial lymphatic sinus (Figure 3d; Shin et al., 2019). This fortuitous observation suggested that a physical structure was hindering the passage of flow between two separate lymphatic compartments. This block in flow was dependent on anesthetic treatment, normally applied to immobilize embryos for angiography and imaging, which prevented musculoskeletal movements. Our subsequent analysis revealed the presence of a lymphatic valve (see details below) at the location of fluid blockage in the facial lymphatic vessel in anesthetized larvae. We also identified a lymphovenous valve at the interface between the rostral end of the facial lymphatic vessel and the primary head sinus, a vessel that carries venous blood flow away from cranial vessels. Since the orientation and position of valves in this location was reminiscent of lymphangion segments in mouse collecting lymphatic vessels, we refer to it as the facial collecting lymphatic vessel (FCLV, Figure 3c–e). Within the facial lymphatic network, lymph drains from a ventrally‐located branch of the facial lymphatic vessel, which extends ventral to the eye, while a dorsal cranial lymphatic branch drains into the caudal region of the FLV. Interestingly, the FLV and FCLV lie in close association with parachordal and hyosymplectic cartilages, which are supported by inner and outer muscle bundles used in lower jaw or gill movements associated with feeding or breathing (Figure 3f). Therefore, we would speculate that these activities would stimulate compression of the FLV to promote lymph transport into the FCLV, with the lymphatic and lymphovenous valves preventing backflow. Consistent with these observations, zebrafish mutant larvae lacking a lymphatic valve, or wild type larvae subjected to long term treatment with anesthetic, show edema around the eye ((Shin et al., 2019), Shin, personal observation).

### 4.2 Molecular identity and structure of the zebrafish lymphatic valve

**FIGURE 4:**
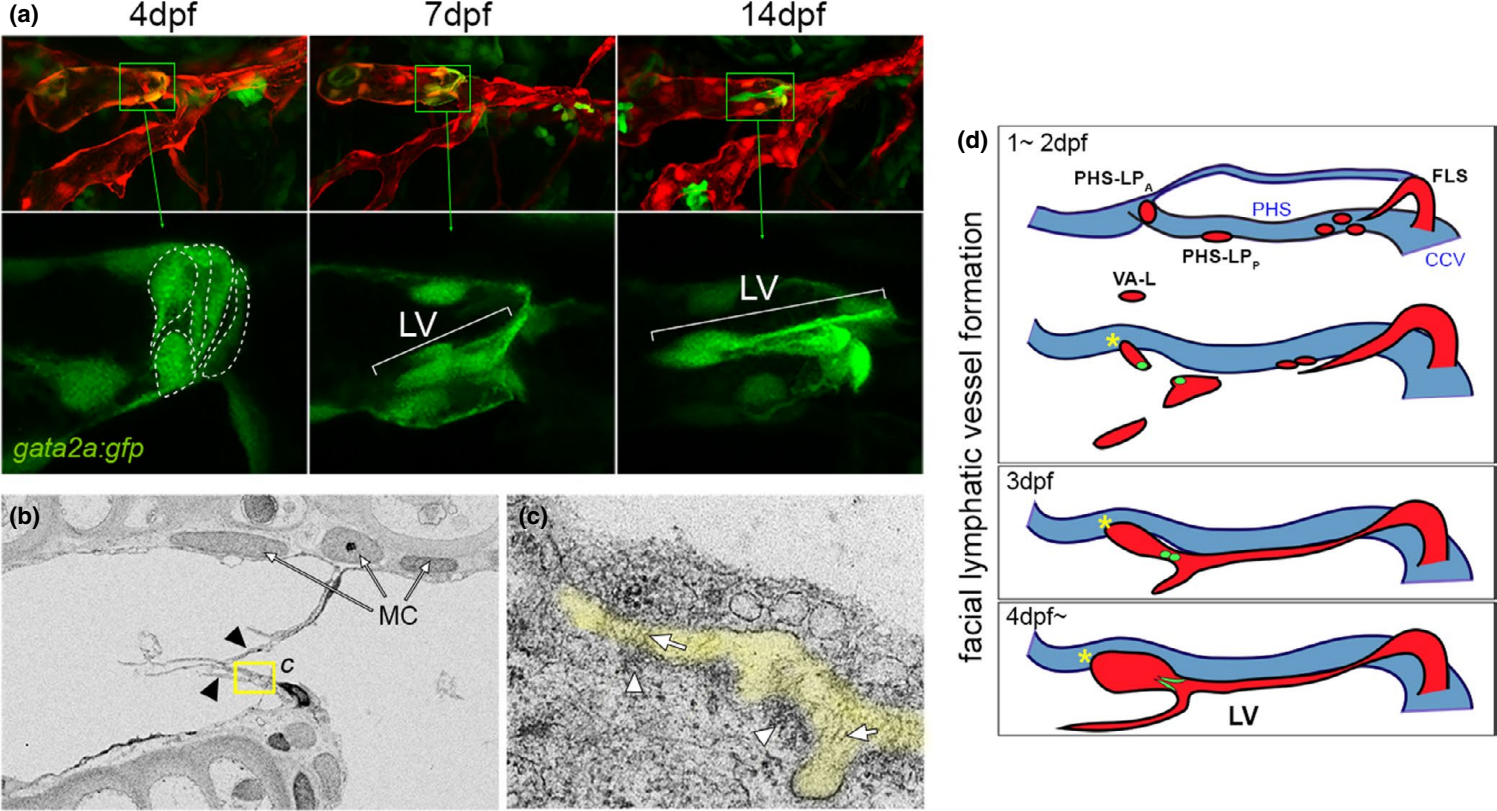
Cellular and ultrastructural features of zebrafish larval lymphatic valves. (a) Confocal images of facial lymphatic vessels at indicated time point. Lymphatic endothelial cells are in red, lymphatic valve (LV) cells also express the *gata2a:gfp* transgene in green. Bottom panels are magnified region indicated in corresponding top image; only green channel is shown. Valve cell shapes traced with dotted lines at 4 dpf. Lateral view, anterior to left, dorsal is up. (b) Scanning electron microgram of lymphatic valve leaflets (indicated by arrowheads). Mural cells (MCs) adjacent to lymphatic endothelial cells are shown. Yellow box indicates region magnified in (c). (c) Extracellular matrix (ECM) core between two lymphatic valve endothelial cells is pseudocolored in yellow. Fibril‐bundles are shown by arrows and electron dense regions suggestive of matrix binding are denoted by arrowheads. (d) Schematic depicting facial lymphatic vessel (red) morphogenesis; primary head sinus (PHS) shown in blue. Lymphatic valve progenitors and valve shown in green. CCV, common cardinal vein; FLS, facial lymphatic sprout; FLV, facial lymphatic vessel; PHS‐LP_A_, primary head sinus‐anterior lymphatic progenitor; PHS‐LP_P_, primary head sinus‐posterior lymphatic progenitor. Images (a–c) are re‐used from Shin et al. (2019) with permission

Molecular characterization of zebrafish LvECs suggested a high degree of similarity to those in mouse. Zebrafish LvECs show specific expression of a *gata2a:egfp* transgene, consistent with the expression of the mammalian Gata2 ortholog (Figure 4a). *Gata2a:egfp* expression initiated prior to valve morphogenesis allowing visualization of cell condensation and formation of a ring‐like structure similar to that seen during morphogenesis in mouse lymphatic valves. By 7 days after fertilization (dpf), *gata2a:egfp* was clearly evident in two intraluminal leaflets within the facial lymphatic vessel (FLV), as well as the lymphovenous valve (LVV in Figure 3e) between the FLV and the primary head sinus. LvEC‐specific expression could also be recapitulated using a highly conserved enhancer element from *gata2a* intron 4. A core sequence in this element is nearly identical in human and mouse, where it is known to bind Gata2 itself and respond to OSS (see above). Whether the zebrafish similarly responds to disturbed flow patterns has not been assessed, but deletion of this endogenous enhancer perturbs normal lymphatic valve development and leads to edema in zebrafish larvae (Shin et al., 2019). In addition to *gata2a:egfp*, we also observed expression of other known mammalian lymphatic valve marker genes in zebrafish LvECs, including zebrafish orthologs of Prox1, *Nfatc1*, *Foxc1* and *Itga9*.

In parallel to live imaging, ultrastructural analysis using electron microscopy revealed step‐wise morphological changes in developing zebrafish lymphatic valves similar to those in mouse. Beginning at 4 dpf, LvEC condensation is apparent, and leaflets subsequently protrude into the intraluminal space from opposing walls to form a nascent bicuspid valve. Transmission EM at 7 dpf and 14 dpf showed that each leaflet comprised a monolayer of cells attached to an extracellular matrix core, with evidence of fibril‐like structures and electron dense regions at the membrane, suggesting attachment points between cells and matrix proteins (Figure 4b,c; Shin et al., 2019). We made similar observations in the FLV of the medaka (*Oryzias latipes*), a related teleost, which diverged from zebrafish (*Danio rerio*) more than 100 million years ago, suggesting that valves may be a shared feature of the teleost lymphatic system (Shin et al., 2019). We have also observed *pdgfrb*‐positive mural cell coverage of the FCLV and FLV, although at larval stages these cells do not express *acta2*, a marker of smooth muscle cell differentiation. Whether these mural cells eventually differentiate into smooth muscle and how they contribute to zebrafish lymphatic function at later stages is currently not known.

### 4.3 Visualizing early steps of lymphatic valve morphogenesis in zebrafish

A major benefit to using the transparent zebrafish embryo is the opportunity to directly visualize the origin of LvEC progenitors as they initiated valvulogenesis. These observations revealed that cells from two distinct locations in the FLV contributed to the developing lymphatic valve. The FLV forms between 2 dpf and 4 dpf by contribution from four distinct venous endothelial sources: the anterior and middle primary head sinus (PHS‐LP_A_, PHS‐LP_P_), common cardinal vein (FLS) and ventral aorta (VA‐L; Figure 4d) between 2 dpf and 3 dpf (Eng et al., 2019; Okuda et al., 2012; Shin et al., 2019). Cells from the PHS‐LP_A_ and ‐LP_P_ migrate laterally to occupy the anterior part of the lateral FLV, while the facial lymphatic sprout (FLS) buds from the common cardinal vein and grows anteriorly to become the posterior section of the lateral FLV. In most cases, primitive lymphatic vessels are venous‐derived. However, a unique population of ventrally located lymphangioblasts (VA‐L) originates from the ventral aorta and migrates dorsally to fuse with the lateral FLV (Eng et al., 2019). Our time‐lapse imaging and lineage tracing revealed that cells from PHS‐LP_A_ mainly contributed to the facial collecting lymphatic vessel and that valve cells originated from both PHS‐LP_A_ and ‐LP_P_. Importantly, the valve appears to form at the location where PHS‐LP_A_ and ‐LP_P_ meet. After their migration from veins, the PHS and FLS progenitors contribute to a formed FLV that appears lumenized at 3 to 4 dpf. At this point, cells in the position of the presumptive valve express *gata2a:gfp* (Shin et al., 2019) indicating specification of lymphatic valve identity. Similar to mouse, we subsequently observed that valve cells become cuboidal and then reorient dorso‐ventrally (perpendicular to the axis of lateral FLV) forming a ring‐like constriction (Figure 4a).

### 4.4 Conserved genetic control of zebrafish lymphatic valve development

Early genetic control of mammalian lymphatic valve formation is governed by a core set of transcription factors that coordinate and control downstream gene expression. These factors include *Prox1*, *Gata2*, *Foxc2*, and *Nfatc1*, all of which are required for early valve formation in the mouse lymphatic system. In our work, we identified similarly essential roles for the zebrafish orthologs of each factor. This work also revealed previously undescribed genetic interactions between these factors during specification and maintenance of LvEC identity (Shin et al., 2019). A brief overview follows.

Prox1 is a master regulatory gene that is essential for the earliest steps of lymphatic specification in mouse and is required for subsequent lymphatic valve development (Hong et al., 2002; Wigle & Oliver, 1999). The zebrafish genome encodes two copies of *prox1*, referred to as *prox1a* and *prox1b*, due to a genome duplication in the teleost lineage. Of these duplicates, *prox1a* is predominantly expressed in the lymphatic system, although loss of both genes together is required to observe significant lymphatic defects similar to those in mouse (Impel et al., 2014). Interestingly, an early description of *prox1a* mutants reported severe lymphedema without an obvious loss in lymphatic morphogenesis. Subsequently, we found that Prox1 expression is pronounced in developing lymphatic valve cells at 4 dpf (Shin et al., 2019). Moreover, *prox1a* mutant larvae fail to initiate *gata2a:gfp* expression in LvECs and do not form lymphatic valves. The distinct phenotypes in *prox1a* and *prox1a*/*prox1b* double mutants suggest there may be different requirements for Prox1 dosage during early specification and LvEC development (Koltowska et al., 2015).

Similar to Prox1, the zebrafish genome also encodes two duplicates of the valve master regulatory transcription factor, Gata2: *gata2a* and *gata2b*. In zebrafish, *gata2a* is expressed in multiple tissues including neurons, sensory organs, pituitary gland and blood vascular endothelial cells. By contrast, *gata2b* is largely restricted to hemogenic endothelium (Andrzejczuk et al., 2018; Butko et al., 2015; Hüsken et al., 2014; Quiroz et al., 2012). As noted above, LvECs express the *TgBAC(gata2a:gfp)^la3^* transgene beginning at 3 dpf. In *gata2a* mutants, early LvECs appear to be specified at 4 dpf, similar to recent observations in Gata2 deficient mouse embryos (Mahamud et al., 2019). However, LvECs in *gata2a* mutants retained a cuboidal shape and failed to form leaflets. Accordingly, *gata2a* mutants exhibit lymphedema at 7 dpf. We observed a similar phenotype in zebrafish bearing a deletion in the conserved intronic endothelial enhancer noted above. In this case, *gata2a* expression was only lost from endothelial cells, suggesting a cell autonomous role for *gata2a* in lymphatic valve morphogenesis, similar to mouse. Notably, a small subset of patients with Emberger syndrome bear deletions in the conserved *GATA2* intron 4 enhancer (Hsu et al., 2013; Spinner et al., 2014), underscoring a conserved role for this enhancer and suggesting that the zebrafish could provide a helpful model for deriving mechanistic insights in these cases.

In mouse, *Foxc1* and *Foxc2* encode closely related forkhead transcription factors with overlapping expression patterns and functions in multiple tissues and are required for cardiovascular development and somitogenesis (Bell et al., 2001; Finegold et al., 2001; Kume et al., 1998). Despite their overlapping function, loss of Foxc2 alone leads to lymphatic valve defects in mouse embryos, while mutations in human *FOXC2* are associated with lymphedema (Fang et al., 2000; Iida et al., 1997; Kume et al., 1998; Winnier et al., 1997). The zebrafish genome lacks a clear *foxc2* ortholog, but possesses duplicated *foxc1* genes, referred to as *foxc1a* and *foxc1b* (Topczewska et al., 2001). Unlike mouse Foxc1 and Foxc2, *foxc1a* alone is required in multiple tissues, with single *foxc1a* mutant zebrafish exhibiting heart defects, circulatory defects, and lymphedema. By rescuing early defects in *foxc1a* mutants, we found that *foxc1a* was also required for lymphatic valve formation. Indeed, FLV LECs failed to express *gata2a:egfp* in *foxc1a* mutants, while LvECs retained a flattened appearance and failed to protrude into the lumen. Interestingly, Norden et al. (2020) found that mouse *Foxc1* function in response to laminar shear stress was distinct from that of *Foxc2* in the mature lymphatic valve (Norden et al., 2020). Thus, it will be of interest to investigate whether *foxc1b* acts in parallel to *foxc1a* in this context.

In addition to the factors noted above, we also identified essential conserved roles for the transcription factor *nfatc1* and the zebrafish ortholog of *Itga9*. In the case of the former, we detected expression of transgene of *nfatc1:gal4ff;uas:kaede* in LECs throughout the FLV prior to lymphatic valve formation (Shin et al., 2019). Furthermore, *SEM* analysis revealed that lymphatic valve cells were absent in *nfatc1* mutants leading to lymphedema. In *itga9* mutant larvae, we likewise noted edema, while early lymphatic specification and morphogenesis appeared normal. Similar to *Itga9* mutant mice, zebrafish lacking *itga9* exhibited disorganized lymphatic valve leaflet structure. Among potential ligands for Itga9 in mammals is Svep1, which is expressed by mesenchymal cells surrounding lymphatic vessels. Mice lacking Svep1 fail to form lymphatic valves, while *svep1* mutant zebrafish show edema around the eyes, gut, and heart, along with mild defects in lymphatic morphogenesis (Karpanen et al., 2017). Whether zebrafish svep1 mutants display lymphatic valve defects is unknown, but our recent work should serve as a means for evaluating this possibility. Taken together, our findings underscore that the genetic program for lymphatic valve formation is largely conserved in the zebrafish.

### 4.5 Related lymphatic structures in other fish species

**FIGURE 5:**
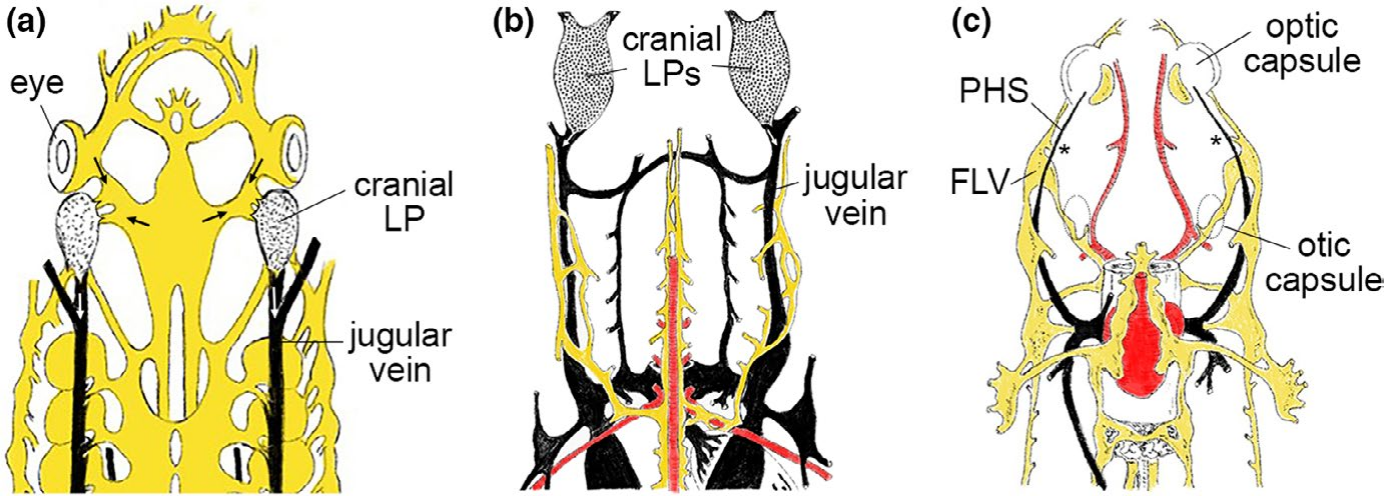
Examples of lymphatic anatomy in cartilaginous and bony fish. Traced drawings depicting the anterior lymphovenous networks in (a) jawless fish (lamprey, after metamorphosis), (b) cartilaginous fish (shark) and (c) bony fish (trout). Dorsal views, lymphatic vessels depicted in yellow, veins in black, and arteries in red. (a, b) Cranial lymph propulsor (LP) and jugular veins are noted. Arrows indicate direction of flow. (c) Primary head sinus (PHS) and facial lymphatic vessel (FLV) assigned based on similar anatomical positions in larval zebrafish. Traced sketches after Kampeier, 1969 with permission

The anatomical location of the FLV in zebrafish is reminiscent of a structure referred to as the lymph propulsor (LP), initially identified in jawless fish (Kampmeier, 1969). In lamprey, paired cranial LPs with a sac‐like structure receive lymph from facial lymphatics and drain into jugular veins through a lymphovenous valve (Figure 5a). Similar observations have also been made in cartilaginous fish (e.g. sharks), although here the LPs were initially thought to comprise a secondary venous system that emptied into the jugular vein (Gegenbaur (1872) and Parker (1887), Figure 5b). Indeed, this secondary venous system in these cases did not seem to be fully separated from venous system due to the appearance of blood cells, suggesting the absence of functional lymphovenous valves among this class of vertebrates. LPs in teleost fish species were also noted in the early 20th century by Allen (1906), with significant variability in size depending on the species. For example, some species of bony fish (e.g. rock‐fish) have remarkable cranial LPs with large sacs while some (e.g. trout) have structures anatomically similar to the LPs of jawless fish and the FLV of zebrafish (Figure 5c). However, early characterization of LPs by dye perfusion failed to clarify whether these structures comprised a network of lymphatic vessels distinct from the circulatory system. Furthermore, valves do not appear to have been documented in cranial LPs of jawless and cartilaginous fishes, although these observations were limited to observations using gross anatomy and classic histology techniques (e.g. dye injection, hematoxylin and eosin stained sections on a low magnification microscope). Our recent studies support the existence of a lymphatic anatomy in both zebrafish and Medaka similar to that of mammals, comprised of distinct compartments separated by valves to facilitate unidirectional fluid return to the circulatory system. As noted above, zebrafish and Medaka diverged more than 100 million years ago and are only two of nearly 30,000 teleost species. Thus, it is likely that collecting lymphatic vessels with valves are a more common feature among this lineage. It will therefore be of interest to determine if this is the case in other model teleost species, including three‐spined stickleback (*Gasterosteus aculeatus*) and killifish (*Fundulus heteroclitus*, *Nothobranchius furzeri*), as well as holostean species including spotted gar (*Lepisosteus oculatus*) and bowfin fish (*Amia calva*).

## 5 CONCLUSION AND FUTURE DIRECTIONS

The lymphatic system has been a subject of general scientific interest for the past 2000 years. Careful anatomical studies throughout this period eventually led to a detailed understanding of how the lymphatic system functioned, including the essential role for lymphatic valves. With the more recent advent of genetic approaches in mouse, we have an increasingly comprehensive description of the molecular pathways that contribute to lymphatic valve formation. Indeed, these studies have revealed a series of discrete cellular processes and associated molecular pathways required for each step of lymphatic valve morphogenesis. Most importantly, these genetic insights have proven clinically relevant for several rare diseases in humans where mutation in genes now known to be required for lymphatic valve development result in lymphedema. Our recent finding of lymphatic valves in the zebrafish, along with the conserved nature of their development, demonstrate that other vertebrate models can contribute to a better understanding of lymphatic valve development. The zebrafish model brings to bear the ability for detailed serial imaging in vivo and amenability to a number of genetic and small molecule screening approaches. These benefits have already begun to reveal new insights into the earliest steps of lymphatic valve formation. Moving forward, the use of the zebrafish to analyze lymphatic valve development will likely contribute to the discovery of new essential genes and provide important mechanistic insights with relevance to related rare human diseases.

## CONFLICT OF INTEREST

The authors declare no competing interests.
